# Causal association between gut microbiota and hyperemesis gravidarum: a two-sample Mendelian randomization study

**DOI:** 10.3389/fmicb.2024.1307729

**Published:** 2024-04-03

**Authors:** Dinglin Xu, Liang Zhang, Jianwei Zhang

**Affiliations:** ^1^The First Clinical Medical College, Shandong University of Traditional Chinese Medicine, Jinan, China; ^2^Reproductive and Genetic Center of Integrated Traditional and Western Medicine, Affiliated Hospital of Shandong University of Traditional Chinese Medicine, Jinan, China

**Keywords:** hyperemesis gravidarum, gut microbiota, causal relationship, Mendelian randomization, *Coprococcus*

## Abstract

**Background:**

Observational studies have reported an association between the gut microbiota (GM) and hyperemesis gravidarum (HG). However, the causal relationship is unclear. In this study, Mendelian randomization (MR) was used to infer causal relationships between GM and HG.

**Methods:**

Inverse-variance weighted MR was performed using summary statistics for genetic variants from genome-wide association studies (GWAS). Sensitivity analyses were performed to validate the MR results and assess the robustness of the causal inference. Reverse MR analysis was performed for bacterial taxa that were causally linked to the HG risk in the forward MR analysis to evaluate reverse causality.

**Results:**

MR analysis revealed that the genera *Defluviitaleaceae UCG011*, *Ruminococcus1*, *Ruminococcus2*, *Turicibacter*, and *unknowngenus* and phylum *Verrucomicrobiota* are positively associated with the risk of HG. Additionally, the genus *Coprococcus2* was related to a decreased risk of HG. Sensitivity studies validated the strength and reliability of the link between the composition of the GM and HG. No evidence for reverse causality from HG to identified bacterial taxa was found.

**Conclusion:**

Our MR analysis provided novel insight into the association between GM and HG. In particular, our results indicated that targeting the GM could serve as an effective therapeutic strategy for HG.

## Introduction

Hyperemesis gravidarum (HG) is characterized by severe nausea, involving more than three episodes of vomiting per day, sufficient to produce >5% weight loss, dehydration, ketosis, alkalosis, hypokalemia, and nutritional disturbances as well as ketones in the urine (Solomon et al., 2023). The primary etiology of HG is an abrupt elevation in serum concentrations of hormones, such as human chorionic gonadotropin (HCG) and estrogen (Jones, 1982). The precise etiology of HG remains uncertain; nevertheless, certain risk factors have been identified, including multiple pregnancies, primigravida, prior history of HG, molar pregnancy, unexpected pregnancy, and family history of HG (Mekonnen et al., 2018). Protective factors include the utilization of multivitamins before 6 weeks of gestational age and maternal cigarette smoking (Czeizel et al., 1992). HG can lead to various clinical symptoms, such as an electrolyte imbalance, malnutrition, muscle weakness, depression, and anxiety in pregnant women and even trigger the development of serious diseases, such as gestational hypertension, Wernicke encephalopathy, and hyperthyroidism syndrome in pregnancy (Jennings and Mahdy, 2023).

There is growing evidence to support an association between the gut microbiota (GM) and HG. The gut microbiota of pregnant women with HG includes a decreased number of beneficial anti-inflammatory species and an increase in pathogenic and opportunistic pathogens, suggesting that the GM plays a role in inflammation in HG (Min, 2020). A study has shown that probiotics reduce Modified Pregnancy-Unique Quantification of Emesis and Nausea (MPUQE) scores without significant adverse effects and can be used as an adjunctive treatment for HG (Xin and Zhenhua, 2021). Changes in the composition of the gut microbiota during pregnancy may be related to the development of nausea and vomiting during pregnancy (Koren et al., 2012).

MR represents an innovative methodology for examining causality (e.g., between GM and HG). In MR, genetic variants are used as instrumental variables (IVs) to establish a causal relationship between exposure and illness outcomes (Greenland, 2000). The random allocation of genotypes from parents to offspring ensures that the correlations between genetic variants and outcomes remain unaffected by common confounding factors, thereby establishing a plausible causal relationship (Burgess and Thompson, 2021). MR has been utilized to investigate the causal relationships between the gut microbiota and many diseases, including metabolic (Sanna et al., 2019), autoimmune (Li C. et al., 2022), and psychiatric disorders (Chen et al., 2022) as well as pregnancy complications (Li C. et al., 2022). In this study, genome-wide association study (GWAS) summary statistics obtained from MiBioGen and the IEU Open GWAS project were used for a two-sample MR analysis to assess the causal relationship between GM and HG.

## Materials and methods

### Data sources

Gut microbiota data were derived from the largest genome-wide meta-analysis available to date published by the MiBioGen consortium (Kurilshikov et al., 2021). The large-scale, multi-ancestry, genome-wide meta-analysis involved 16S ribosomal RNA (rRNA) gene sequencing profiles and genotyping data for 18,340 participants from 24 population cohorts of European, Middle Eastern, East Asian, American Hispanic/Latin, and African American origin to examine associations between autosomal human genetic variants and the gut microbiome. The microbial composition was analyzed by targeting three variable regions of the 16 S rRNA gene: V4, V3–V4, and V1–V2. Genetic loci that influence the relative abundance (microbiome quantitative trait loci) or presence (microbiome binary trait loci) of microbial taxa were identified by using microbiome trait loci mapping. The results included 211 taxa (131 genera, 35 families, 20 orders, 16 classes, and 9 phyla). The genus was the lowest taxonomic rank evaluated. HG data were obtained from a GWAS dataset from 2021 in FinnGen Biobank, which included 1,148 cases and 110,330 controls of European ancestry. In total, 16,379,549 single nucleotide polymorphisms (SNPs) were analyzed. The data can be downloaded from the IEU OpenGWAS project.^1^

### Two-sample MR design

**Figure 1:**
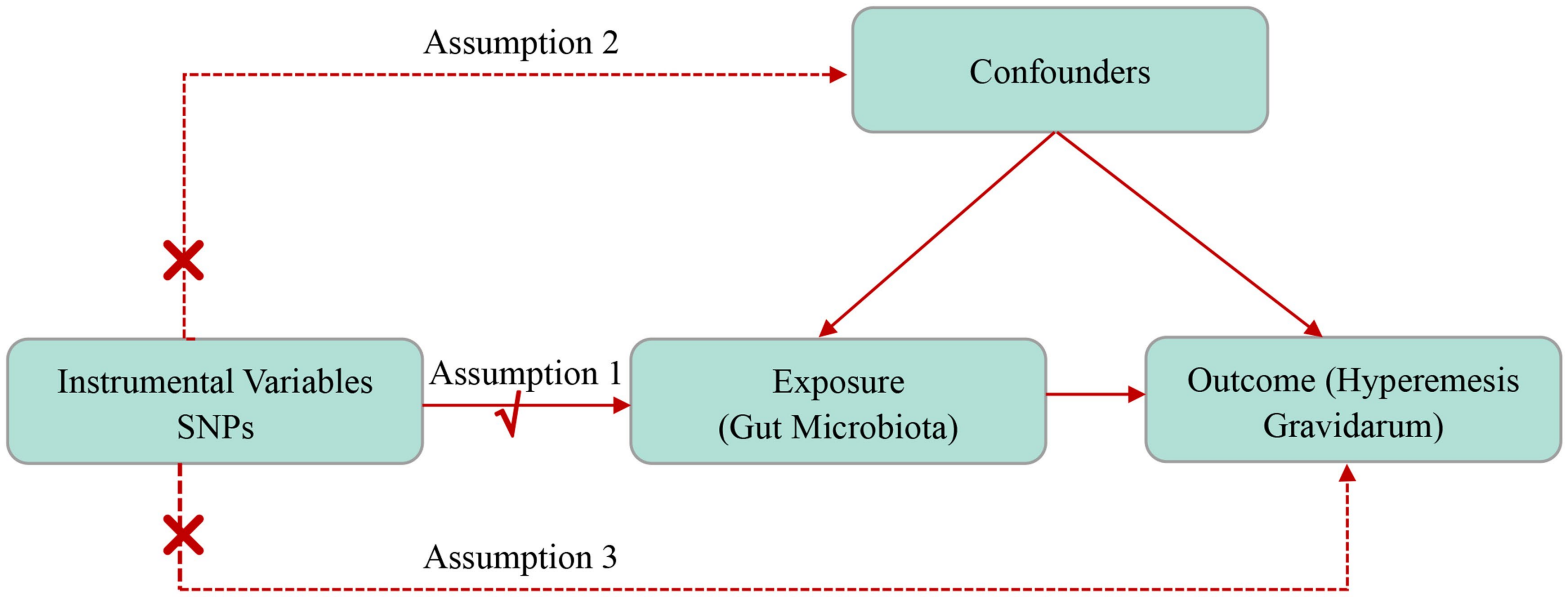
The study design of the present MR study.

The MR framework requires three assumptions (Figure 1) (1) genetic variants as IVs are robustly associated with exposure factors (the relevance assumption); (2) IVs are not associated with any potential confounders (the independence assumption); (3) the genetic variant only affects outcomes through the exposure (the exclusion restriction assumption). Figure 1 depicts a concise description of the directional MR design.

### Selection of IVs

The following quality control measures were used to choose the most suitable IVs to ensure the reliability and precision of the results regarding the link between GM and HG. (1) Two thresholds were employed in the selection of IVs. The initial IVs were SNPs with a significance of lower than the genome-wide threshold of 5 × 10^−8^. However, only a small number of IVs were obtained. For a broader investigation of the connections between severe vomiting in pregnancy and GM, a significance level of 1 × 10^−5^ was also used to identify SNPs. (2) SNPs in linkage disequilibrium (LD) were removed (*R*^2^ < 0.001 and clumping window size = 10,000 kb). (3) Palindromic SNPs (e.g., with A/T or G/C alleles) were removed. (4) The strength of IVs was evaluated by calculating *F*-statistics, where an *F*-statistic exceeding 10 indicated that a weak IV bias is highly unlikely. *F*-statistics were calculated as follows:


$$F=\left[\left(N-K-1\right)/K\right]\times R^{2}/\left(1-R^{2}\right)$$

*R^2^* and *N* refer to the cumulative variance explained by the selected SNPs and sample size, respectively, and *K* is the number of SNPs. The formula for *R^2^* was as follows:


$$R^{2}=\frac{2\times \beta^{2}\times EAF\times \left(1-EAF\right)}{2\times \beta^{2}\times EAF\times \left(1-EAF\right)+2\times {SE}^{2}\times N\times EAF\times \left(1-EAF\right)}$$

where EAF is the effect allele frequency, *β* is the allele effect value, and SE is the standard error.

### MR analysis

To investigate whether there is a causal relationship between the GM and excessive vomiting in pregnancy, five widely utilized Mendelian randomization (MR) techniques were used: inverse-variance weighted (IVW) (Burgess et al., 2013), weighted (Hartwig et al., 2017), MR-Egger regression (Bowden et al., 2015), weighted median estimator (WME) (Bowden et al., 2016), and simple. The IVW technique has slightly higher statistical power than those of alternative methods under specific circumstances (Bowden et al., 2016). Consequently, when multiple IVs were utilized, the primary analysis predominantly relied on the IVW method, while the remaining four methods were employed as supplementary approaches. Bidirectional MR was used to rule out causal effects of HG on GM. A reverse MR analysis was performed only for bacterial genera with a significant causal relationship with HG in forward analyses.

### Sensitivity analysis

To assess the robustness of the results, sensitivity analyses were performed, including heterogeneity and pleiotropy tests. Cochran’s *Q* test was used to assess the heterogeneity in the effects of each SNP. In addition, to identify potentially heterogeneous SNPs, a leave-one-out sensitivity test was conducted by systematically eliminating each SNP individually. The intercept from the MR-Egger tests was used to detect horizontal pleiotropy in multiple IVs (Burgess and Thompson, 2017). A *p*-value of less than 0.05 indicated heterogeneity.

## Results

### Selection of IVs related to GM

Initially, 211 taxa and 2,246 SNPs were extracted under a locus-wide significance threshold of *p* < 1 × 10^−5^. These taxa included 9 phyla (102 SNPs), 16 classes (178 SNPs), 20 orders (215 SNPs), 35 families (382 SNPs), and 131 genera (1,372 SNPs) after quality control processes, including the removal of LD effects and palindromic SNPs. The *F*-statistics for the IVs were all over 10, indicating a lack of weak instrument bias.

### Two-sample MR analysis

#### MR results at five levels

**Figure 2:**
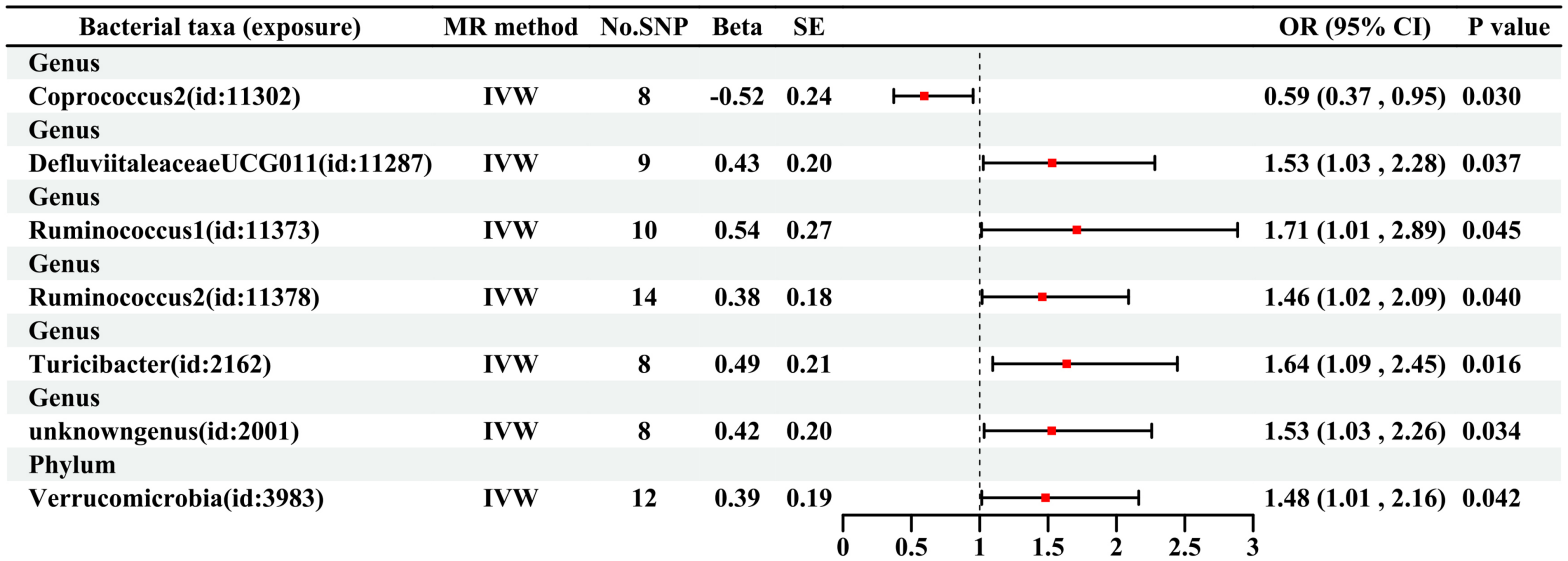
MR estimates according to the IVW method for the association between GM and HG Note: Abbreviations: MR Mendelian randomization, SNP single nucleotide polymorphism, SE Standard error, OR odds ratio, CI confidence interval, IVW inverse variance weighted.

**Figure 3:**
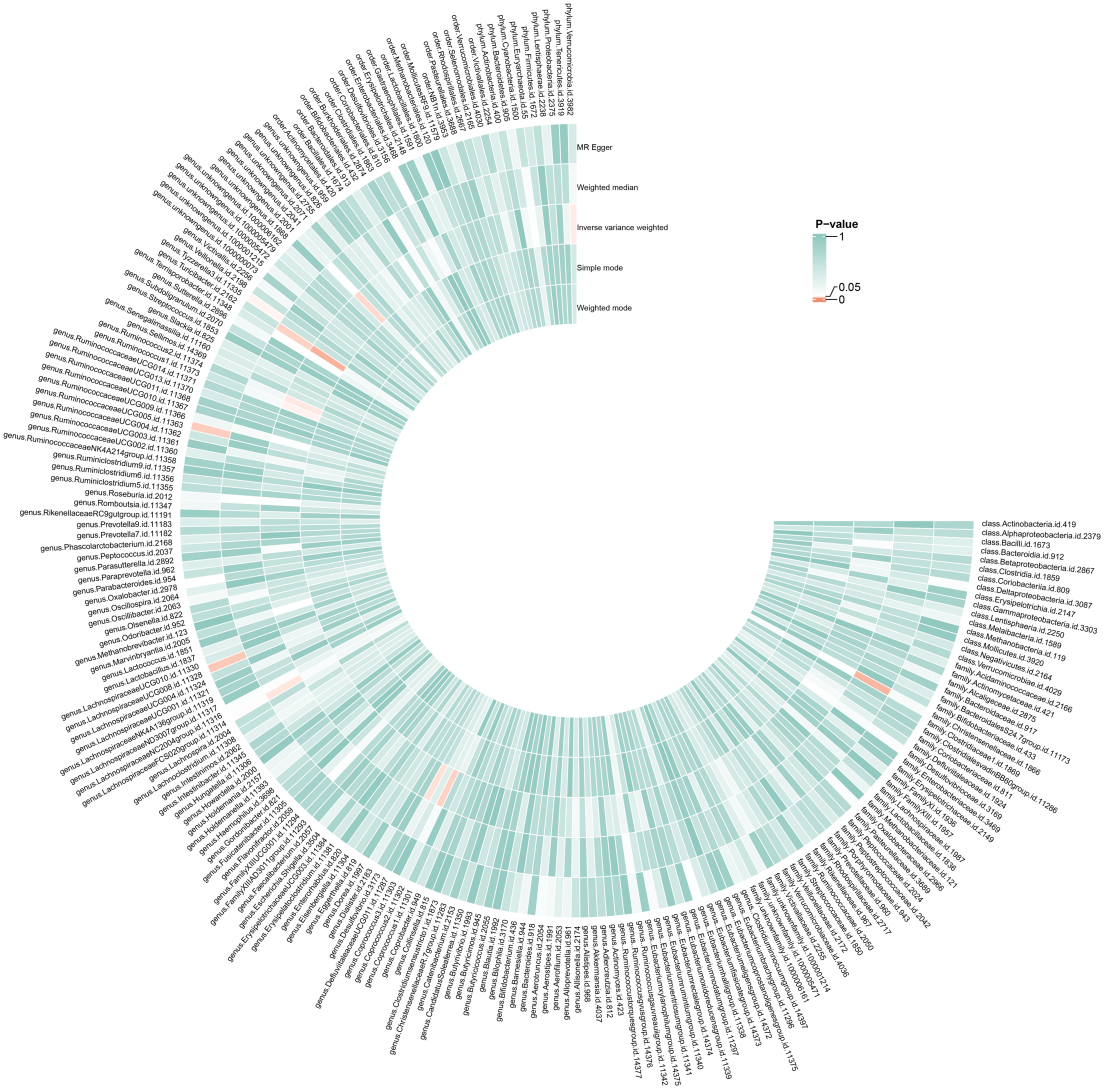
Causal effects of GM on HG. From the inner circle to the outer circle, different statistical methods are represented: Weighted mode, Simple mode, Inverse variance weighted, Weighted median, and MR Egger.

**Figure 4:**
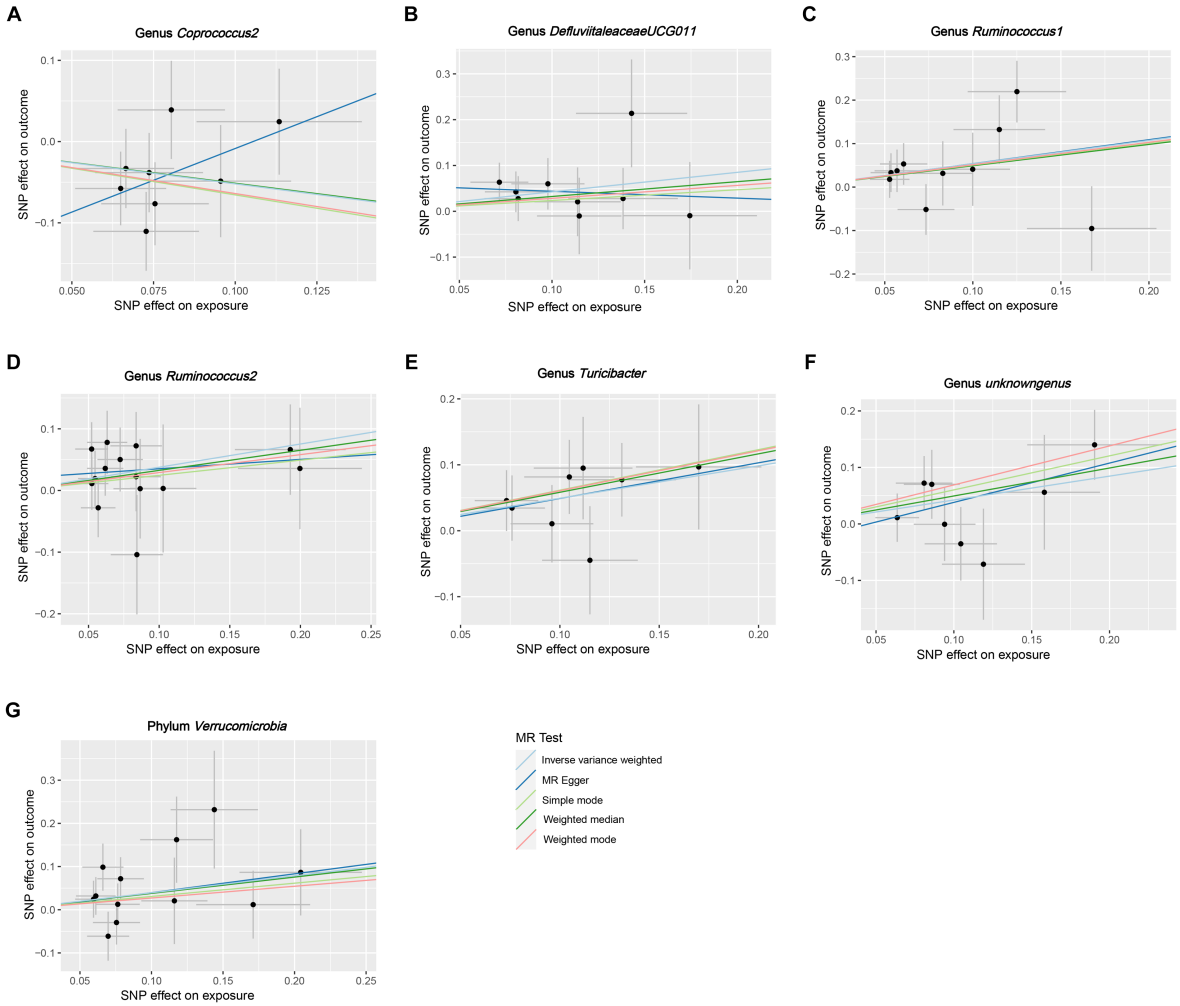
Scatter plots for the causal association between GM and HG. **(A)** Genus *Coprococcus2,* **(B)** Genus *DefluviitaleaceaeUCG011,* **(C)** Genus *Ruminococcus1,* **(D)** genus *Ruminococcus2,* **(E)** Genus *Turicibacter,* **(F)** Genus *unknowngenus,* **(G)** Phylum *Verrucomicrobia*.

As illustrated in Figure 2, seven bacterial taxa were linked to the risk of HG, as determined using the IVW method. In particular, the IVW analysis showed that the genus *DefluviitaleaceaeUCG011* (OR, 1.53; 95% CI, 1.03–2.28; *p* = 0.037), genus *Ruminococcus1* (OR, 1.71; 95% CI, 1.01–2.89; *p* = 0.045), genus *Ruminococcus2* (OR, 1.46; 95% CI, 1.02–2.09; *p* = 0.040), genus *Turicibacter* (OR, 1.64; 95% CI, 1.09–2.45; *p* = 0.016), genus *unknowngenus* (OR, 1.53; 95% CI, 1.03–2.26; *p* = 0.034), and phylum *Verrucomicrobiota* (OR, 1.48; 95% CI, 1.01–2.16; *p* = 0.042) were positively associated with the risk of HG. The relative abundance of the genus *Coprococcus2* (OR: 0.59, 95%CI: 0.37–0.95, *p* = 0.030) was negatively related to the risk of HG. The results for the five methods are presented in Figures 3,4 and Supplementary Table S1.

#### Assessment of assumptions

Cochran’s *Q* test for IVW results indicated that there was no statistically significant heterogeneity among the IVs (Supplementary Table S2). Furthermore, the MR-Egger regression intercepts (Supplementary Table S3) indicated the absence of any substantial directional horizontal pleiotropy. A leave-one-out sensitivity analysis showed that the risk estimations associated with specific bacterial taxa and the risk of HG could not be attributed to a single SNP (Supplementary Figure S1).

### Reverse MR analysis

We performed a reverse MR analysis to infer whether there was a causal link between HG and the relative abundance of seven bacterial taxa. We selected SNPs (*p* < 1 × 10^−5^) that were significantly associated with the risk of HG as IVs. We excluded SNPs in LD (*R*^2^ < 0.1 and clumping window size = 10,000 kb). The other methods and settings were consistent with those used in the forward MR analysis. We did not detect reverse causal relationships between HG and identified bacterial features (Supplementary Table S4). According to Cochran’s *Q* test based on IVW, there was no significant heterogeneity across the included IVs (Supplementary Table S5). The MR-PRESSO global test and the MR-Egger intercept analysis did not reveal discernible horizontal pleiotropy (Supplementary Table S6). A leave-one-out sensitivity analysis showed that the risk estimations associated with the risk of HG and specific bacterial taxa could not be attributed to a single SNP (Supplementary Figure S2).

## Discussion

To investigate the causal relationship between GM and HG, we used a two-sample MR analysis. To the best of our knowledge, this is the first study of the association between GM and HG at the genetic level. Our analysis utilized the most extensive and up-to-date GWAS data available. The results provide valuable insights for the prevention, treatment, and overall management of HG, supporting the targeted modulation of certain GM. In particular, the genus *Coprococcus2* confers protective effects, whereas the genera *DefluviitaleaceaeUCG011*, *Ruminococcus1*, *Ruminococcus2*, *Turicibacter*, and *unknowngenus* and phylum *Verrucomicrobiota* have detrimental effects on HG. Reverse MR analyses did not reveal any evidence of reverse causality from HG to the identified bacterial genera.

MR is not sensitive to confounding effects due to the random allocation of germline genetic variation during meiosis, reflecting exposure without being influenced by reverse causation. The results of our monodirectional, two-sample MR study suggested that genetic predispositions to the genera *DefluviitaleaceaeUCG011* (OR=1.53), *Ruminococcus1* (OR = 1.71), *Ruminococcus2* (OR = 1.46), *Turicibacter* (OR = 1.64), and *unknowngenus* (OR = 1.53) and phylum *Verrucomicrobiota* (OR = 1.48) are associated with 53%, 71%, 46%, 64%, 53%, and 48% elevated risks of HG, respectively. Genetically predicted *Coprococcus2* (OR = 0.59) was associated with a 41% decreased risk of HG.

Dysbiosis of the gut microbiota contributes to various disorders during pregnancy, such as gestational diabetes (Liu et al., 2022), preeclampsia-eclampsia (Li P. et al., 2022), and severe intrahepatic cholestasis of pregnancy (Zhan et al., 2021), and is considered a contributing factor to severe vomiting in pregnancy. HG is characterized by severe nausea and vomiting beginning before the 22nd week of pregnancy (Balci et al., 2022). The association between GM and HG can be attributed to the impact of specific microbial taxa on inflammation and the regulation of sex hormones, either via direct or indirect effects (Jeong et al., 2017). Determining the roles of GH in the regulation of sex hormones, inflammation, immune-related cytokine production, and the modulation of metabolic pathways, thereby impacting HG, is an important research topic (Adlercreutz et al., 1984). Previous research has focused on individuals diagnosed with HG based on data from fecal samples. However, it is important to note that a cross-sectional study design makes it impossible to establish a causal link between the gut microbiota and HG (Min, 2020). We utilized MR to examine the association between GM and HG in human subjects, enabling us to control for confounding factors and establish a reliable causal link.

Among taxa associated with HG in this study, *Ruminococcus* is a gram-positive, anaerobic, spherical bacterium that does not produce spores. It is associated with autism spectrum disorder, along with *Streptococcus* and *Ruminiclostridium* (Wang et al., 2023). There is evidence that *Ruminococcus* can partially inhibit inflammation and protect the intestinal mucosa after cisplatin treatment in diverse cancer models (Perales-Puchalt et al., 2018; Hsiao et al., 2021). Numerous studies have demonstrated a potential correlation between a reduction in *Turicibacter* and the occurrence of colitis. This reduction has been linked to elevated levels of tumor necrosis factor and NF-κB1 as well as lower levels of butyric acid in the intestines (Jones-Hall et al., 2015; Zhong et al., 2015; Liu et al., 2016). The prevalence of the relatively unexplored bacterium *Turicibacter* was notably reduced in the gastrointestinal microbiota of C57BL/6 mice and APC+/1,638 N mice with high-fat diet-induced obesity; this reduction in the abundance of *Turicibacter* was associated with heightened levels of inflammation and activation of the Wnt-signaling pathway (Liu et al., 2016; Guo et al., 2017). These findings were contrary to those of our study, in which *Turicibacter* was associated with an elevated risk of HG. Additionally, a link between *Verrucomicrobiota* and HG has not been reported. Hence, our research suggests a novel avenue for investigating the role of the gut microflora and the mechanisms by which these bacteria contribute to HG. We detected a significant negative association between *Coprococcus2* and HG. Several studies have shown a decrease in the prevalence of *Coprococcus* in individuals suffering from sepsis (Yu et al., 2022). It is possible that *Coprococcus* modulates inflammatory dynamics in the host (Lin et al., 2023). A reduced prevalence of this bacterial lineage may lead to an increased responsiveness to inflammatory stimuli in the host, thereby exacerbating the severity of sepsis. In pregnant women with HG, *Coprococcus2* was a protective factor, possibly via reducing inflammatory responses, providing a bases for the development of therapeutic strategies.

Our study had several strengths. To the best of our knowledge, this is the first application of MR to assess the causal relationship between GM and HG based on extensive GWAS data. Unlike prior observational studies, the MR approach mitigates potential biases, such as confounding variables and reverse causation, thereby improving the accuracy of causal inferences. Furthermore, various sensitivity analyses and Cochran’s Q statistics revealed a lack of heterogeneity or pleiotropy among the IVs, supporting the precision and reliability of the findings.

However, this study had some limitations. First, GWAS are unlikely to reveal all genetic traits associated with complex phenotypes (Altshuler et al., 2008). Understanding the hereditary basis of a disease can provide a basis for preventive strategies; however, environmental variables often contribute to disease development (Meisel et al., 2015). MR reduces interference from confounding factors to a limited extent. Second, because the GWAS data were only based on people of European ancestry, considering the problem of population stratification, the results may not be generalizable to non-European populations, and more studies of GM and HG are needed verify the results. Third, owing to the small number of IVs meeting the strict criteria (*p* < 5 × 10^−8^), IVs were screened using a flexible threshold (*p* < 1 × 10^−5^), and there may be some unavoidable confounding factors.

## Conclusion

The genus *Coprococcus2* had protective effects against HG, whereas various genera (i.e., *DefluviitaleaceaeUCG011*, *Ruminococcus1*, *Ruminococcus2*, *Turicibacter*, and *unknowngenus*) and the phylum *Verrucomicrobiota* had detrimental effects. Experimental studies are necessary to explore the mechanisms by which specific gut microbial taxa influence HG. The observed relationships between GM and HG suggest that alterations in the diversity and composition of the intestinal flora can be used in clinical diagnosis, prevention, and treatment, providing a basis for further research on gynecological diseases. In the future, we can also explore the mechanisms by which specific gut microbiota influence HG to further provide a basis for the treatment of HG. Despite a lack of support for reverse causality, the potential impact of HG on the composition of the gut microbiota cannot be ruled out. Additional studies are needed to confirm the relationships.

## Data availability statement

Publicly available datasets were analyzed in this study. This data can be found here: these data were derived from the following resources available in the public websites: the exposure data for gut microbiota https://mibiogen.gcc.rug.nl/, and the outcome data for hyperemesis gravidarum https://gwas.mrcieu.ac.uk/.

## Author contributions

DX: Data curation, Validation, Formal Analysis, Writing - original draft. LZ: Conceptualization, Visualization, Writing - review & editing. JZ: Methodology, Supervision, Writing - review & editing.

## Footnotes

^1^ https://gwas.mrcieu.ac.uk/

## References

[ref1] Adlercreutz H. Pulkkinen M. O. Hämäläinen E. K. Korpela J. T. (1984). Studies on the role of intestinal bacteria in metabolism of synthetic and natural steroid hormones. J. Steroid Biochem. 20, 217–229. doi: 10.1016/0022-4731(84)90208-5, PMID: 6231418

[ref2] Altshuler D. Daly M. J. Lander E. S. (2008). Genetic mapping in human disease. Science 322, 881–888. doi: 10.1126/science.1156409, PMID: 18988837 PMC2694957

[ref3] Balci S. Tohma Y. A. Esin S. Onalan G. Tekindal M. A. Zeyneloglu H. B. (2022). Gut dysbiosis may be associated with hyperemesis gravidarum. J. Matern. Fetal Neonatal Med. 35, 2041–2045. doi: 10.1080/14767058.2020.1777268, PMID: 32519907

[ref4] Bowden J. Davey Smith G. Burgess S. (2015). Mendelian randomization with invalid instruments: effect estimation and bias detection through egger regression. Int. J. Epidemiol. 44, 512–525. doi: 10.1093/ije/dyv080, PMID: 26050253 PMC4469799

[ref5] Bowden J. Davey Smith G. Haycock P. C. Burgess S. (2016). Consistent estimation in Mendelian randomization with some invalid instruments using a weighted median estimator. Genet. Epidemiol. 40, 304–314. doi: 10.1002/gepi.21965, PMID: 27061298 PMC4849733

[ref6] Burgess S. Thompson S. G.. (2021). "Mendelian randomization: methods for causal inference using genetic variants." CRC Press, New York

[ref7] Burgess S. Butterworth A. Thompson S. G. (2013). Mendelian randomization analysis with multiple genetic variants using summarized data. Genet. Epidemiol. 37, 658–665. doi: 10.1002/gepi.21758, PMID: 24114802 PMC4377079

[ref8] Burgess S. Thompson S. G. (2017). Interpreting findings from Mendelian randomization using the MR-egger method. Eur. J. Epidemiol. 32, 377–389. doi: 10.1007/s10654-017-0255-x, PMID: 28527048 PMC5506233

[ref9] Chen M. Xie C. R. Shi Y. Z. Tang T. C. Zheng H. (2022). Gut microbiota and major depressive disorder: a bidirectional Mendelian randomization. J. Affect. Disord. 316, 187–193. doi: 10.1016/j.jad.2022.08.012, PMID: 35961601

[ref10] Czeizel A. E. Dudas I. Fritz G. Técsöi A. Hanck A. Kunovits G. (1992). The effect of periconceptional multivitamin-mineral supplementation on vertigo, nausea and vomiting in the first trimester of pregnancy. Arch. Gynecol. Obstet. 251, 181–185. doi: 10.1007/bf02718384, PMID: 1503509

[ref11] Greenland S. (2000). An introduction to instrumental variables for epidemiologists. Int. J. Epidemiol. 29, 722–729. doi: 10.1093/ije/29.4.722, PMID: 10922351

[ref12] Guo X. Li J. Tang R. Zhang G. Zeng H. Wood R. J.. (2017). High fat diet alters gut microbiota and the expression of Paneth cell-antimicrobial peptides preceding changes of circulating inflammatory cytokines. Mediat. Inflamm. 2017, 9474896–9474899. doi: 10.1155/2017/9474896, PMID: 28316379 PMC5339499

[ref13] Hartwig F. P. Davey Smith G. Bowden J. (2017). Robust inference in summary data Mendelian randomization via the zero modal pleiotropy assumption. Int. J. Epidemiol. 46, 1985–1998. doi: 10.1093/ije/dyx102, PMID: 29040600 PMC5837715

[ref14] Hsiao Y. P. Chen H. L. Tsai J. N. Lin M. Y. Liao J. W. Wei M. S.. (2021). Administration of *Lactobacillus reuteri* combined with *Clostridium butyricum* attenuates cisplatin-induced renal damage by gut microbiota reconstitution, increasing butyric acid production, and suppressing renal inflammation. Nutrients 13:2792. doi: 10.3390/nu13082792, PMID: 34444952 PMC8402234

[ref15] Jennings L. K. Mahdy H. (2023). Hyperemesis gravidarum, Treasure Island, FL, StatPearls Publishing LLC. 30422512

[ref16] Jeong S. Y. Kang S. Hua C. S. Ting Z. Park S. (2017). Synbiotic effects of β-glucans from cauliflower mushroom and *Lactobacillus fermentum* on metabolic changes and gut microbiome in estrogen-deficient rats. Genes Nutr. 12:31. doi: 10.1186/s12263-017-0585-z, PMID: 29151980 PMC5679333

[ref17] Jones G. W. (1982). “Obstetrics and gynaecology in Tudor and Stuart England”. Essay review. Trans. Stud. Coll. Physicians Phila. 4, 313–318. PMID: 7179433

[ref18] Jones-Hall Y. L. Kozik A. Nakatsu C. (2015). Correction: ablation of tumor necrosis factor is associated with decreased inflammation and alterations of the microbiota in a mouse model of inflammatory bowel disease. PLoS One 10:e0125309. doi: 10.1371/journal.pone.0125309, PMID: 25860670 PMC4393316

[ref19] Koren O. Goodrich J. K. Cullender T. C. Spor A. Laitinen K. Bäckhed H. K.. (2012). Host remodeling of the gut microbiome and metabolic changes during pregnancy. Cell 150, 470–480. doi: 10.1016/j.cell.2012.07.008, PMID: 22863002 PMC3505857

[ref20] Kurilshikov A. Medina-Gomez C. Bacigalupe R. Radjabzadeh D. Wang J. Demirkan A.. (2021). Large-scale association analyses identify host factors influencing human gut microbiome composition. Nat. Genet. 53, 156–165. doi: 10.1038/s41588-020-00763-1, PMID: 33462485 PMC8515199

[ref21] Li C. Liu C. Li N. (2022). Causal associations between gut microbiota and adverse pregnancy outcomes: a two-sample Mendelian randomization study. Front. Microbiol. 13:1059281. doi: 10.3389/fmicb.2022.1059281, PMID: 36590417 PMC9801412

[ref22] Li P. Wang H. Guo L. Gou X. Chen G. Lin D.. (2022). Association between gut microbiota and preeclampsia-eclampsia: a two-sample Mendelian randomization study. BMC Med. 20:443. doi: 10.1186/s12916-022-02657-x, PMID: 36380372 PMC9667679

[ref23] Lin T. C. Soorneedi A. Guan Y. Tang Y. Shi E. Moore M. D.. (2023). Turicibacter fermentation enhances the inhibitory effects of *Antrodia camphorata* supplementation on tumorigenic serotonin and Wnt pathways and promotes ROS-mediated apoptosis of Caco-2 cells. Front. Pharmacol. 14:1203087. doi: 10.3389/fphar.2023.1203087, PMID: 37663253 PMC10469317

[ref24] Liu W. Crott J. W. Lyu L. Pfalzer A. C. Li J. Choi S. W.. (2016). Diet-and genetically-induced obesity produces alterations in the microbiome, inflammation and Wnt pathway in the intestine of Apc^+/1638N^ mice: comparisons and contrasts. J. Cancer 7, 1780–1790. doi: 10.7150/jca.15792, PMID: 27698916 PMC5039360

[ref25] Liu K. Zou J. Fan H. Hu H. You Z. (2022). Causal effects of gut microbiota on diabetic retinopathy: a Mendelian randomization study. Front. Immunol. 13:930318. doi: 10.3389/fimmu.2022.930318, PMID: 36159877 PMC9496187

[ref26] Meisel S. F. Beeken R. J. van Jaarsveld C. H. Wardle J. (2015). Genetic susceptibility testing and readiness to control weight: results from a randomized controlled trial. Obesity 23, 305–312. doi: 10.1002/oby.20958, PMID: 25522302 PMC4361051

[ref27] Mekonnen A. Amogne F. K. Kassahun C. W. (2018). Risk factors of hyperemesis gravidarum among pregnant women in bale zone hospitals, Southeast Ethiopia: unmatched case-control study. Clin. Mother Child Health 15:2. doi: 10.4172/2090-7214.1000300

[ref28] Min J. (2020). “Study on the relationship between intestinal flora and gastrointestinal dysfunction during pregnancy” in Doctoral thesis (Jinan city, Shandong province:Shangdong University) doi: 10.27272/d.cnki.gshdu.2020.000166

[ref29] Perales-Puchalt A. Perez-Sanz J. Payne K. K. Svoronos N. Allegrezza M. J. Chaurio R. A.. (2018). Frontline science: microbiota reconstitution restores intestinal integrity after cisplatin therapy. J. Leukoc. Biol. 103, 799–805. doi: 10.1002/jlb.5hi1117-446rr, PMID: 29537705 PMC6004318

[ref30] Sanna S. van Zuydam N. R. Mahajan A. Kurilshikov A. Vich Vila A. Võsa U.. (2019). Causal relationships among the gut microbiome, short-chain fatty acids and metabolic diseases. Nat. Genet. 51, 600–605. doi: 10.1038/s41588-019-0350-x, PMID: 30778224 PMC6441384

[ref31] Solomon D. Morka G. Wayessa Z. J. (2023). Determinants of hyperemesis gravidarum among pregnant women in public hospitals of Guji, West Guji, and Borana zones, Oromia, Ethiopia, 2022. SAGE Open Med. 11:20503121231196713. doi: 10.1177/20503121231196713, PMID: 37701795 PMC10493065

[ref32] Wang H. Liu S. Xie L. Wang J. (2023). Gut microbiota signature in children with autism spectrum disorder who suffered from chronic gastrointestinal symptoms. BMC Pediatr. 23:476. doi: 10.1186/s12887-023-04292-8, PMID: 37730588 PMC10510216

[ref33] Xin X. Zhenhua W. (2021). Clinical value of probiotics and prebiotics in the adjuvant treatment of hyperemesis gravidarum. Chin. J. Fam. Plan. Gynecotokol. 13, 72–75. doi: 10.3969/j.issn.1674-4020.2021.07.21

[ref34] Yu J. Li H. Zhao J. Huang Y. Liu C. Yang P.. (2022). Alterations of the gut microbiome in Chinese Zhuang ethnic patients with Sepsis. Mediat. Inflamm. 2022:2808249. doi: 10.1155/2022/2808249, PMID: 35633656 PMC9142305

[ref35] Zhan Q. Qi X. Weng R. Xi F. Chen Y. Wang Y.. (2021). Alterations of the human gut microbiota in intrahepatic cholestasis of pregnancy. Front. Cell. Infect. Microbiol. 11:635680. doi: 10.3389/fcimb.2021.635680, PMID: 33996622 PMC8120235

[ref36] Zhong Y. Nyman M. Fåk F. (2015). Modulation of gut microbiota in rats fed high-fat diets by processing whole-grain barley to barley malt. Mol. Nutr. Food Res. 59, 2066–2076. doi: 10.1002/mnfr.201500187, PMID: 26184884

