# Supplementary material for: Causal association between gut microbiota and hyperemesis gravidarum: a two-sample Mendelian randomization study
Source: Front Microbiol. 2024 Apr 3;15:1307729. doi: 10.3389/fmicb.2024.1307729 (PMC11021657; doi:10.3389/fmicb.2024.1307729)
Supplement: Supplementary file 1 [file Table_1.docx]

Supplementary Material

# Supplementary Tables

Supplementary Table S1 MR estimates for the association between GM and HG.

| Bacterial taxa (exposure) | MR method | No.  SNP | Beta | SE | OR | 95%CI | P-value |
| --- | --- | --- | --- | --- | --- | --- | --- |
| genus Coprococcus2 | MR Egger | 8 | 1.57 | 1.39 | 4.79 | 0.31-72.84 | 0.303 |
|  | Weighted median | 8 | -0.51 | 0.34 | 0.60 | 0.31-1.16 | 0.131 |
|  | IVW | 8 | -0.52 | 0.24 | 0.59 | 0.37-0.95 | 0.030 |
|  | Simple mode | 8 | -0.66 | 0.52 | 0.52 | 0.19-1.44 | 0.249 |
|  | Weighted mode | 8 | -0.64 | 0.53 | 0.53 | 0.19-1.48 | 0.263 |
| genus DefluviitaleaceaeUCG011 | MR Egger | 9 | -0.15 | 0.78 | 0.86 | 0.19-3.97 | 0.853 |
|  | Weighted median | 9 | 0.32 | 0.26 | 1.38 | 0.82-2.32 | 0.222 |
|  | IVW | 9 | 0.43 | 0.20 | 1.53 | 1.03-2.28 | 0.037 |
|  | Simple mode | 9 | 0.24 | 0.38 | 1.27 | 0.60-2.66 | 0.551 |
|  | Weighted mode | 9 | 0.28 | 0.35 | 1.33 | 0.67-2.63 | 0.443 |
| genus Ruminococcus1 | MR Egger | 10 | 0.55 | 0.77 | 1.74 | 0.38-7.88 | 0.496 |
|  | Weighted median | 10 | 0.49 | 0.33 | 1.63 | 0.85-3.14 | 0.142 |
|  | IVW | 10 | 0.54 | 0.27 | 1.71 | 1.01-2.89 | 0.045 |
|  | Simple mode | 10 | 0.51 | 0.61 | 1.66 | 0.51-5.46 | 0.425 |
|  | Weighted mode | 10 | 0.52 | 0.68 | 1.67 | 0.44-6.38 | 0.470 |
| genus Ruminococcus2 | MR Egger | 14 | 0.15 | 0.43 | 1.16 | 0.50-2.71 | 0.732 |
|  | Weighted median | 14 | 0.33 | 0.25 | 1.39 | 0.85-2.27 | 0.195 |
|  | IVW | 14 | 0.38 | 0.18 | 1.46 | 1.02-2.09 | 0.040 |
|  | Simple mode | 14 | 0.25 | 0.36 | 1.28 | 0.63-2.59 | 0.506 |
|  | Weighted mode | 14 | 0.29 | 0.30 | 1.34 | 0.75-2.39 | 0.344 |
| genus Turicibacter | MR Egger | 8 | 0.54 | 0.82 | 1.72 | 0.35-8.51 | 0.532 |
|  | Weighted median | 8 | 0.58 | 0.27 | 1.79 | 1.06-3.02 | 0.028 |
|  | IVW | 8 | 0.49 | 0.21 | 1.64 | 1.09-2.45 | 0.016 |
|  | Simple mode | 8 | 0.61 | 0.38 | 1.85 | 0.88-3.88 | 0.147 |
|  | Weighted mode | 8 | 0.61 | 0.33 | 1.83 | 0.96-3.52 | 0.111 |
| genus unknowngenus | MR Egger | 8 | 0.70 | 0.54 | 2.01 | 0.70-5.76 | 0.244 |
|  | Weighted median | 8 | 0.49 | 0.27 | 1.64 | 0.96-2.80 | 0.070 |
|  | IVW | 8 | 0.42 | 0.20 | 1.53 | 1.03-2.26 | 0.034 |
|  | Simple mode | 8 | 0.60 | 0.47 | 1.83 | 0.72-4.63 | 0.245 |
|  | Weighted mode | 8 | 0.69 | 0.33 | 1.99 | 1.05-3.79 | 0.073 |
| phylum Verrucomicrobia | MR Egger | 12 | 0.44 | 0.51 | 1.55 | 0.57-4.17 | 0.408 |
|  | Weighted median | 12 | 0.38 | 0.26 | 1.46 | 0.88-2.43 | 0.145 |
|  | IVW | 12 | 0.39 | 0.19 | 1.48 | 1.01-2.16 | 0.042 |
|  | Simple mode | 12 | 0.31 | 0.40 | 1.36 | 0.62-3.00 | 0.462 |
|  | Weighted mode | 12 | 0.27 | 0.34 | 1.31 | 0.67-2.56 | 0.442 |

Note: Abbreviations: MR Mendelian randomization, SNP single nucleotide polymorphism, SE Standard error, OR odds ratio, CI confidence interval, IVW inverse variance weighted.

Supplementary Table S2 The heterogeneity of GM instrumental variables.

| Bacterial taxa (exposure) | Method | Cochran's Q | df | P-value |
| --- | --- | --- | --- | --- |
| genus Coprococcus2 | MR Egger | 4.16 | 6 | 0.65 |
|  | IVW | 6.49 | 7 | 0.48 |
| genus DefluviitaleaceaeUCG011 | MR Egger | 3.23 | 7 | 0.86 |
|  | IVW | 3.82 | 8 | 0.87 |
| genus Ruminococcus1 | MR Egger | 11.82 | 8 | 0.16 |
|  | IVW | 11.82 | 9 | 0.22 |
| genus Ruminococcus2 | MR Egger | 6.34 | 12 | 0.90 |
|  | IVW | 6.67 | 13 | 0.92 |
| genus Turicibacter | MR Egger | 2.59 | 6 | 0.86 |
|  | IVW | 2.59 | 7 | 0.92 |
| genus unknowngenus | MR Egger | 5.11 | 6 | 0.53 |
|  | IVW | 5.41 | 7 | 0.61 |
| phylum Verrucomicrobia | MR Egger | 9.97 | 10 | 0.44 |
|  | IVW | 9.98 | 11 | 0.53 |

Supplementary Table S3 Directional horizontal pleiotropy assessed by intercept term in MR Egger regression of the association between GM and HG.

| Bacterial taxa (exposure) | Egger intercept | SE | P-value |
| --- | --- | --- | --- |
| genus Coprococcus2 | -0.17 | 0.11 | 0.18 |
| genus DefluviitaleaceaeUCG011 | 0.06 | 0.08 | 0.47 |
| genus Ruminococcus1 | 0.00 | 0.06 | 0.98 |
| genus Ruminococcus2 | 0.02 | 0.03 | 0.57 |
| genus Turicibacter | -0.01 | 0.08 | 0.95 |
| genus unknowngenus | -0.03 | 0.06 | 0.61 |
| phylum Verrucomicrobia | 0.00 | 0.04 | 0.93 |

Supplementary Table S4 MR estimates for the association between HG and GM.

| Bacterial taxa (outcome) | MR method | No. SNP | Beta | SE | OR | 95%CI | P-value |
| --- | --- | --- | --- | --- | --- | --- | --- |
| genus Coprococcus2 | MR Egger | 16 | 0.04 | 0.05 | 1.04 | 0.94-1.15 | 0.497 |
|  | Weighted median | 16 | 0.01 | 0.02 | 1.01 | 0.97-1.06 | 0.544 |
|  | IVW | 16 | 0.00 | 0.02 | 1.00 | 0.97-1.03 | 0.900 |
|  | Simple mode | 16 | 0.00 | 0.04 | 1.00 | 0.93-1.08 | 0.958 |
|  | Weighted mode | 16 | 0.01 | 0.03 | 1.01 | 0.94-1.08 | 0.750 |
| genus DefluviitaleaceaeUCG011 | MR Egger | 16 | 0.06 | 0.06 | 1.07 | 0.95-1.19 | 0.286 |
|  | Weighted median | 16 | -0.01 | 0.03 | 0.99 | 0.94-1.04 | 0.749 |
|  | IVW | 16 | 0.00 | 0.02 | 1.00 | 0.96-1.04 | 0.990 |
|  | Simple mode | 16 | -0.03 | 0.05 | 0.97 | 0.89-1.07 | 0.578 |
|  | Weighted mode | 16 | -0.02 | 0.05 | 0.98 | 0.90-1.07 | 0.723 |
| genus Ruminococcus1 | MR Egger | 16 | 0.01 | 0.04 | 1.01 | 0.93-1.10 | 0.769 |
|  | Weighted median | 16 | 0.00 | 0.02 | 1.00 | 0.96-1.04 | 0.989 |
|  | IVW | 16 | 0.00 | 0.01 | 1.00 | 0.98-1.03 | 0.766 |
|  | Simple mode | 16 | 0.00 | 0.03 | 1.00 | 0.94-1.07 | 0.884 |
|  | Weighted mode | 16 | 0.00 | 0.03 | 1.00 | 0.94-1.05 | 0.950 |
| genus Ruminococcus2 | MR Egger | 16 | -0.01 | 0.04 | 0.99 | 0.91-1.08 | 0.835 |
|  | Weighted median | 16 | 0.01 | 0.02 | 1.01 | 0.97-1.05 | 0.599 |
|  | IVW | 16 | 0.02 | 0.01 | 1.02 | 1.00-1.05 | 0.090 |
|  | Simple mode | 16 | -0.01 | 0.03 | 0.99 | 0.93-1.06 | 0.835 |
|  | Weighted mode | 16 | 0.00 | 0.03 | 1.00 | 0.95-1.06 | 0.934 |
| genus Turicibacter | MR Egger | 16 | 0.08 | 0.06 | 1.08 | 0.97-1.21 | 0.173 |
|  | Weighted median | 16 | 0.02 | 0.03 | 1.02 | 0.97-1.07 | 0.422 |
|  | IVW | 16 | 0.03 | 0.02 | 1.03 | 0.99-1.06 | 0.122 |
|  | Simple mode | 16 | 0.03 | 0.05 | 1.03 | 0.94-1.12 | 0.559 |
|  | Weighted mode | 16 | 0.01 | 0.04 | 1.01 | 0.93-1.11 | 0.781 |
| genus unknowngenus | MR Egger | 16 | 0.08 | 0.06 | 1.08 | 0.97-1.21 | 0.173 |
|  | Weighted median | 16 | 0.02 | 0.02 | 1.02 | 0.97-1.07 | 0.401 |
|  | IVW | 16 | 0.03 | 0.02 | 1.03 | 0.99-1.06 | 0.122 |
|  | Simple mode | 16 | 0.03 | 0.05 | 1.03 | 0.94-1.13 | 0.570 |
|  | Weighted mode | 16 | 0.01 | 0.05 | 1.01 | 0.92-1.11 | 0.791 |
| phylum Verrucomicrobia | MR Egger | 16 | 0.07 | 0.05 | 1.07 | 0.97-1.17 | 0.178 |
|  | Weighted median | 16 | 0.01 | 0.02 | 1.01 | 0.97-1.05 | 0.720 |
|  | IVW | 16 | -0.01 | 0.02 | 0.99 | 0.96-1.02 | 0.692 |
|  | Simple mode | 16 | 0.03 | 0.04 | 1.03 | 0.95-1.12 | 0.439 |
|  | Weighted mode | 16 | 0.03 | 0.04 | 1.04 | 0.96-1.12 | 0.397 |

Note: MR Mendelian randomization, SNP single nucleotide polymorphism, SE Standard error, OR odds ratio, CI confidence interval, IVW inverse variance weighted

Supplementary Table S5 The heterogeneity of HG instrumental variables.

| Bacterial taxa (outcome) | Method | Cochran's Q | df | P-value |
| --- | --- | --- | --- | --- |
| genus Coprococcus2 | MR Egger | 15.29 | 14 | 0.359 |
|  | IVW | 15.95 | 15 | 0.385 |
| genus DefluviitaleaceaeUCG011 | MR Egger | 9.49 | 14 | 0.799 |
|  | IVW | 10.85 | 15 | 0.763 |
| genus Ruminococcus1 | MR Egger | 14.81 | 14 | 0.391 |
|  | IVW | 14.86 | 15 | 0.462 |
| genus Ruminococcus2 | MR Egger | 12.11 | 14 | 0.597 |
|  | IVW | 12.75 | 15 | 0.621 |
| genus Turicibacter | MR Egger | 12.12 | 14 | 0.596 |
|  | IVW | 13.10 | 15 | 0.594 |
| genus unknowngenus | MR Egger | 12.12 | 14 | 0.596 |
|  | IVW | 13.10 | 15 | 0.594 |
| phylum Verrucomicrobia | MR Egger | 9.75 | 14 | 0.780 |
|  | IVW | 12.42 | 15 | 0.647 |

Note: MR Mendelian randomization, IVW inverse variance weighted

Supplementary Table S6 Directional horizontal pleiotropy assessed by intercept term in MR Egger regression of the association between HG and GM.

| Bacterial taxa (outcome) | Egger intercept | SE | P-value |
| --- | --- | --- | --- |
| genus Coprococcus2 | -0.01 | 0.01 | 0.449 |
| genus DefluviitaleaceaeUCG011 | -0.02 | 0.01 | 0.263 |
| genus Ruminococcus1 | 0.00 | 0.01 | 0.831 |
| genus Ruminococcus2 | 0.01 | 0.01 | 0.438 |
| genus Turicibacter | -0.01 | 0.01 | 0.339 |
| genus unknowngenus | -0.01 | 0.01 | 0.339 |
| phylum Verrucomicrobia | -0.02 | 0.01 | 0.125 |

Note: SE Standard error

# Supplementary Figures


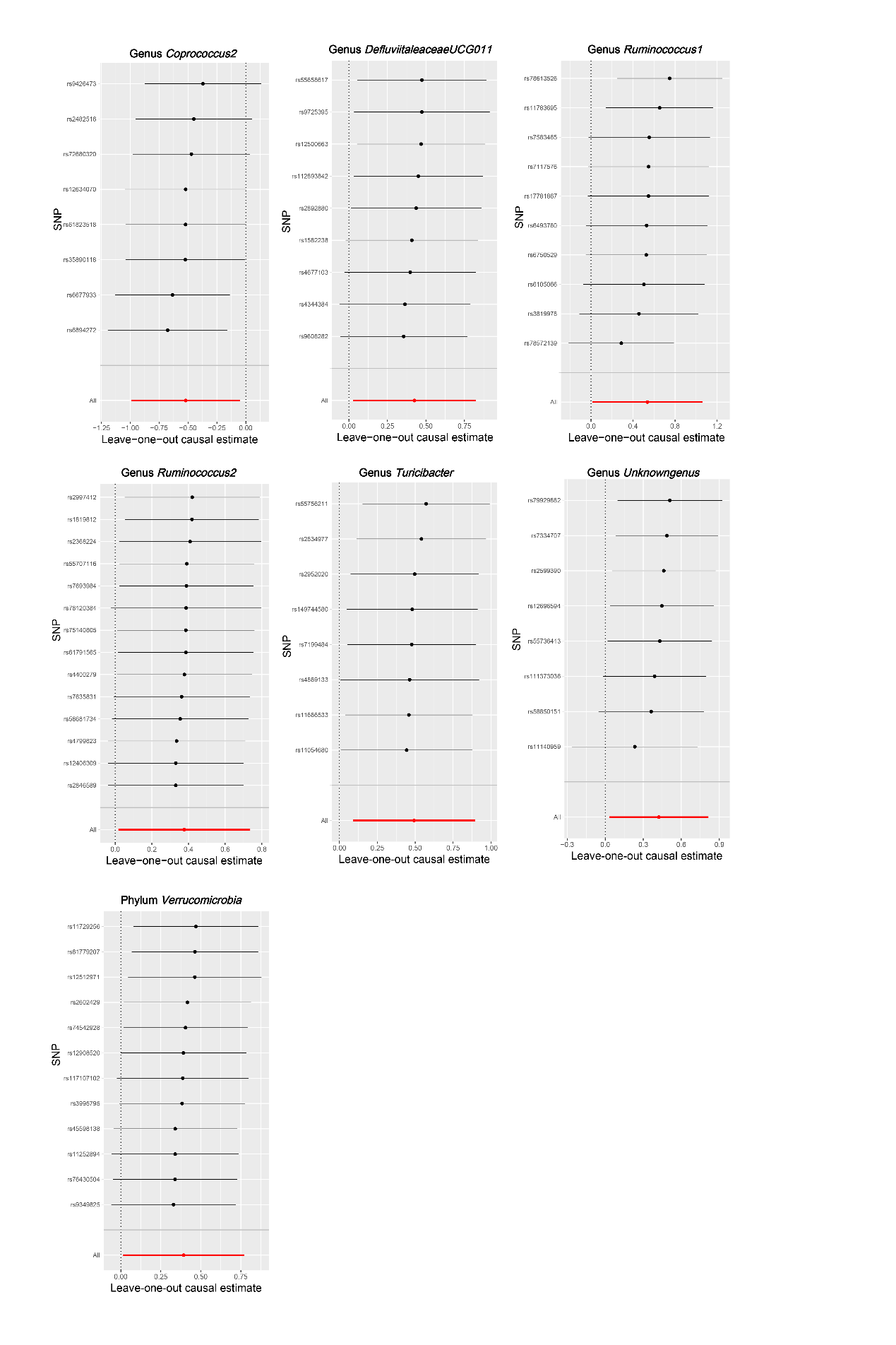


Supplementary Figure S 1 Leave-one-out plots for causal effects of GM on HG.


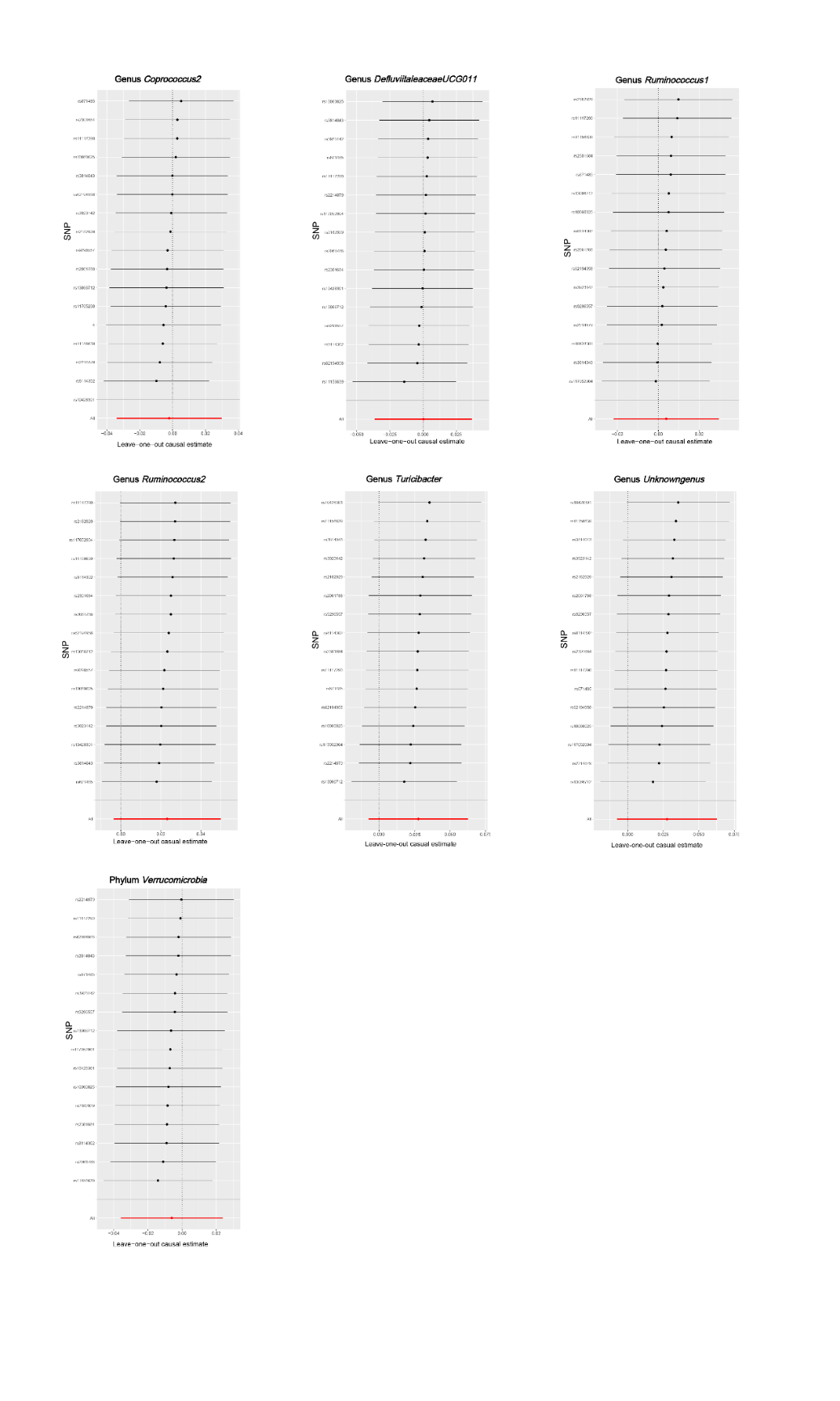


Supplementary Figure S 2 Leave-one-out plots for causal effects of HG on GM.
